# New global minimum conformers for the Pt$_{19}$ and Pt$_{20}$ clusters: low symmetric species featuring different active sites

**DOI:** 10.1007/s00894-024-06099-5

**Published:** 2024-08-17

**Authors:** José Manuel Guevara-Vela, Miguel Gallegos, Tomás Rocha-Rinza, Álvaro Muñoz-Castro, Peter L. Rodríguez Kessler, Ángel Martín Pendás

**Affiliations:** 1 Departamento de Química Física Aplicada, Universidad Autónoma de Madrid, C. Francisco Tomás y Valiente, 7, Madrid, 28049 Spain; 2 Departamento de Química Física y Analítica, Universidad de Oviedo, Av. Julián Clavería, 8, Oviedo, 33006 Asturias Spain; 3 Instituto de Química, Universidad Nacional Autónoma de México, Circuito Exterior, Ciudad Universitaria, Delegación Coyoacán, 04510 Mexico City Mexico; 4 Facultad de Ingeniería, Arquitectura y Diseño, Universidad San Sebastián, Bellavista 7, Santiago, 8420524 RM Chile; 5 Centro de Investigaciones en Óptica A.C., Loma del Bosque 115, Col. Lomas del Campestre, León, 37150 Guanajuato Mexico

**Keywords:** Metal clusters, Platinum clusters, DFT, Hybrid functionals

## Abstract

****Context**:**

The study of platinum (Pt) clusters and nanoparticles is essential due to their extensive range of potential technological applications, particularly in catalysis. The electronic properties that yield optimal catalytic performance at the nanoscale are significantly influenced by the size and structure of Pt clusters. This research aimed to identify the lowest-energy conformers for Pt$_{18}$, Pt$_{19}$, and Pt$_{20}$ species using Density Functional Theory (DFT). We discovered new low-symmetry conformers for Pt$_{19}$ and Pt$_{20}$, which are 3.0 and 1.0 kcal/mol more stable, respectively, than previously reported structures. Our study highlights the importance of using density functional approximations that incorporate moderate levels of exact Hartree-Fock exchange, alongside basis sets of at least quadruple-zeta quality. The resulting structures are asymmetric with varying active sites, as evidenced by sigma hole analysis on the electrostatic potential surface. This suggests a potential correlation between electronic structure and catalytic properties, warranting further investigation.

****Methods**:**

An equivariant graph neural network interatomic potential (NequIP) within the Atomic Simulation Environment suite (ASE) was used to provide initial geometries of the aggregates under study. DFT calculations were performed with the ORCA 5 package, using functional approximations that included Generalized Gradient Approximation (PBE), meta-GGA (TPSS, M06-L), hybrid (PBE0, PBEh), meta-GGA hybrid (TPSSh), and range-separated hybrid ($\omega$B97x) functionals. Def2-TZVP and Def2-QZVP as well as members of the cc-pwCVXZ-PP family to check basis set convergence were used. QTAIM calculations were performed using the AIMAll suite. Structures were visualized with the AVOGADRO code.

**Supplementary Information:**

The online version contains supplementary material available at 10.1007/s00894-024-06099-5.

## Introduction

Platinum nanoparticles are highly versatile species that find application in a wide range of fields due to their electrochemical [1, 2] and optical properties  [3, 4]. Their importance is even more pronounced in the field of catalysis [5, 6] and especially for the photocatalytic production of H$_2$  [7–10]. Indeed, the importance of hydrogen gas production can hardly be overemphasized, being central to various industrial sectors, including oil refining and steel processing. Furthermore, achieving a global increase in the production of H$_2$ is essential for the decarbonisation of heavy industry, long-haul transportation, and seasonal energy storage  [11]. In this context, the catalytic activity of small Pt clusters outperforms that of larger nanoparticles and bulkier metals due to their unique structures and electronic properties  [12, 13]. Understanding the reasons behind these outstanding characteristics could potentially lead to the development of cheaper and more abundant catalysts, thereby advancing sustainable hydrogen production and helping to reduce our dependence on fossil fuels in the fight against climate change  [14].

This background has given rise to a wide interest in acquiring a comprehensive understanding of the electronic properties and structure of small Pt nanoparticles, whose catalytic performance is strongly influenced by their shape and size  [15]. Consequently, significant effort has been invested in identifying the most thermodynamically stable structures of different small platinum clusters. Despite the high computational cost associated with Density Functional Theory (DFT) calculations in comparison to other methodologies such as the Gupta [16] or Sutton-Chen [17] model potentials, they have been the preferred approach for this endeavor. For instance, Kumar and Kawazoe  [18], on one hand, and Wei and Liu  [19], on the other, conducted systematic investigations using the PBE functional and projected augmented wave (PAW) pseudopotentials to identify isomers of up to 44 and 46 atoms, respectively. These studies have indicated that the addition of single atoms to existing clusters may prove an effective method for identifying novel minimum structures [20]. The search for the most stable structures of these moieties has employed a wide range of techniques, including biased searches  [21], prescreening using empirical potentials  [22], genetic algorithms  [22, 23], simulated annealing  [24], or a combination of them. Moreover, specific studies have focused on finding the global minimum (GM) of specific individual clusters, such as Pt$_{13}$  [21, 25–28], Pt$_{15}$  [22, 24], and Pt$_{55}$  [26, 29, 30]. The use of different functionals or methodologies may result in varying conformer orderings, and this has occasionally given rise to controversies. This underscores the necessity for highly reliable results, which can be achieved by considering a number factors, including the use of large and flexible enough basis sets [31] or of appropriate aproximate exchange-correlation functionals that incorporate a suitable amount of Hartree-Fock exchange  [32].

With this in mind, we employed a simulated annealing procedure based on molecular dynamics simulations, using a machine learning potential trained from DFT calculations, to investigate the structures of Pt$_{18}$–Pt$_{20}$. This approach led to the discovery of new and asymmetric stable minima for Pt$_{19}$ and Pt$_{20}$. While the structure of the new minimum for Pt$_{19}$ is similar to the previously reported ones, the new minimum for Pt$_{20}$ differs significantly from previously accounted structures. Furthermore, our analysis incorporates different DFT approaches and emphasizes the importance of incorporating moderate amounts of Hartree-Fock exchange and the use of large basis sets to accurately determine the energetic ordering of platinum clusters. Finally, employing the Quantum Theory of Atoms in Molecules (QTAIM), we have reveiled the differences in the electronic distribution of the two lowest isomers of the Pt$_{20}$ cluster. We believe that the insights presented in this article will (i) provide important guidelines for the design of future simulations of these systems and (ii) contribute to the accurate energetic ordering of isomers leading to the identification of bona fide GM of metallic clusters.

## Results and discussion

**Fig. 1:**
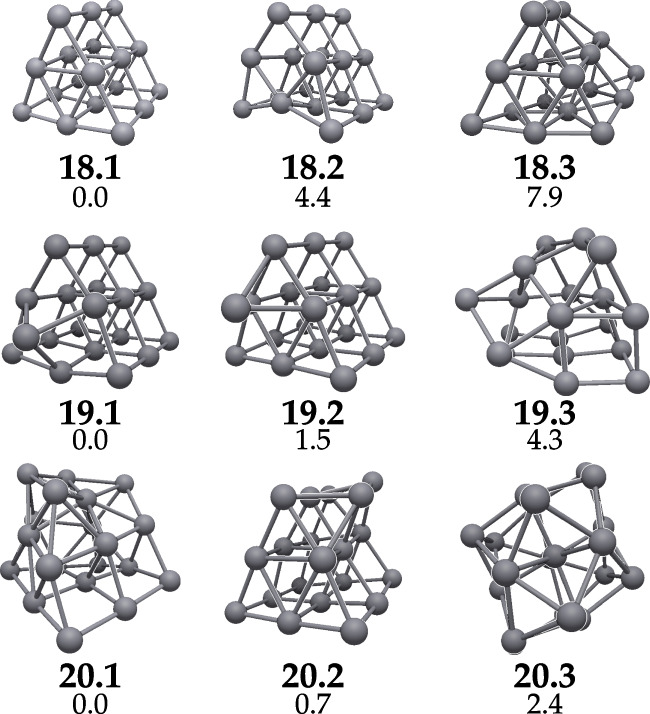
Lowest-lying energy isomers of Pt$_{18}$, Pt$_{19}$, and Pt$_{20}$ along with their labels and relative energy in kcal/mol at the TPSS/Def2-QZVPP//TPSS/Def2-TZVP level of theory

Figure 1 depicts the structures of the lowest-lying energy isomers of Pt$_{18}$, Pt$_{19}$, and Pt$_{20}$. The xyz files corresponding to each structure at the TPSSh level of theory are available as Supporting Information. For Pt$_{18}$, we confirm that the GM, **18**.**1**, is the three-layer trigonal prism structure with D$_{3{\textrm{h}}}$ symmetry proposed by Kumar and Kawazoe [18]. Furthermore, an analogous structure, slightly higher in energy, was identified wherein a Pt atom is displaced in an upward direction from the base, creating a rectangular opening in one of the sides of the pyramid. This results in C$_s$ symmetry. The third Pt$_{18}$ isomer depicted in Fig. 1 exhibits a distorted three-layer trigonal prism structure with C$_2$ symmetry. It is noteworthy that this isomer is identified as the GM when described with the M06-L and $\omega$B97x functionals. As will be discussed in greater detail below, meta-GGA functionals tend to disfavor the three-layer trigonal prism structure. Despite repeated attemps, it was not possible to optimize the **18**.**1** isomer using the $\omega$B97x functional.

It is notable that the use of functional approximations that partially incorporate exact Hartree-Fock exchange results in a reduction in the energy differences between the **18**.**1** and **18**.**2** isomers. Indeed, the aforementioned discrepancy is 4.4 kcal/mol for the TPSS functional, while it is only 0.4 kcal/mol for the TPSSh, which includes 10% HF exchange. Furthermore, going from the PBE to the PBE0 functional reverses the energetic order of the isomers. With PBE, **18**.**1** is the lowest energy isomer, while **18**.**2** is the GM for PBE0, a hybrid functional with 25% exact HF exchange. Indeed, it is established that the inclusion of HF exchange can be pivotal in ensuring the correct energetic ordering [33]. This is because it mitigates the many-electron self-interaction error. This error arises from the inability of approximate exchange-correlation functionals to exclude the interaction of an electron with itself. Consequently, the inclusion of exact exchange facilitates a more precise characterization of electronic systems  [34, 35].

In the case of Pt$_{19}$, a novel GM, designated as the **19**.**1** structure, was identified that exhibits subtle differences from the previously reported minima, **19**.**2**. Both structures are derived from the addition of a Pt atom to one of the triangular sides of the Pt$_{18}$ three-layer trigonal prism (**18**.**1**). In the case of **19**.**2**, the atom is added to one of the interstitial spaces between three other Pt atoms, resulting in a structure with C$_s$ symmetry. With regard to the **19**.**1** isomer, the additional atom is located at an equal distance from the three sides of the triangular face of the three-layer trigonal prism, thereby resulting in C$_{3v}$ symmetry. The third reported isomer, **19**.**3**, exhibits no symmetry elements beyond the identity element. Regarding the energetic order, in this case, there is a more uniform assessment from the different functionals: PBE, PBE0, TPSS, and TPSSh all favor the **19**.**1** structure as the GM. In contrast, and similarly to what happens with Pt$_{18}$, the M06-L and $\omega$B97x functionals predict disordered structures over those containing a three-layer trigonal prism, with a difference in energy of over 10 kcal/mol. As was the case with **18**.**1**, it was not possible to optimize **19**.**1** using the $\omega$B97x functional.

For Pt$_{20}$, the minimum energy structure proposed in previous works [18, 19, 22] (**20**.**2**) is a three-layer trigonal prism with two platinum atoms appended to one of its sides. In contrast, our proposed GM (**20**.**1**) possesses C$_{1}$ symmetry and can be better described as a square base formed by nine Pt atoms, with a second and third levels formed by six and five Pt atoms, respectively. It appears that this unstructured arrangement favors the number of connecting atoms over the directionality of connections, which may indicate the initial stages of the transition from ordered nanoclusters to a metallic arrangement. The third isomer of Pt$_{20}$ put forward in Fig. 1, **20**.**3**, is formed by a rhomboidal-like base with nine atoms with two additional levels on top containing seven and four atoms, respectively. At first glance, its symmetry appears to be C$_2$-like; however, the presence of minor discrepancies between the edges destroys this pseudo-symmetry, leaving **20**.**3** as an additional C$_1$ isomer. Energetically, the TPSS, TPSSh, and $\omega$B97x functionals render **20**.**1** as the GM. The PBE0 functional yields quasi-degenerate energies for the **20**.**1** and **20**.**2** isomers, and PBE favors the latter by 3.1 kcal/mol. Ultimately, optimization of the **20**.**2** isomer was not feasible when using the M06-L functional. Regarding the energetic ordering of the other two isomers, **20**.**3** sits 5.4 kcal/mol lower in energy. Notice, however, that the M06-L **20**.**3** structure has C$_2$ symmetry (Table 1).

**Table 1:** Relative energies (kcal/mol) of the three lowest-lying isomers of Pt18, Pt19, and Pt20 computed with different exchange-correlation functionals as well as the corresponding multiplicity

Label	M	PBE	PBEh	PBE0	TPSS	TPSSh	TPSS0	M06-L	$\omega$B97x
18.1$^{a}$	9	0.0	0.0	5.4	0.0	0.0	7.1	12.2	—
18.2	5	5.1	4.9	0.0	4.4	0.4	0.0	12.9	8.2
18.3	9	13.2	13.2	7.4	7.9	4.6	3.6	0.0	0.0
19.1	9	0.0	0.0	0.0	0.0	0.0	0.0	13.5	—
19.2$^{a}$	9	2.3	2.5	4.7	1.5	3.0	2.8	12.8	13.6
19.3	3	7.0	7.5	6.5	4.3	4.3	0.6	0.0	0.0
20.1	5	3.1	3.3	0.0	0.0	0.0	1.0	5.4	0.0
20.2$^{a}$	5	0.0	0.0	0.0	0.7	1.0	0.0	—	9.1
20.3	3	7.3	7.9	2.7	2.4	3.4	9.0	0.0	23.1

$$^{\text {a}}$$ Ref. [18]

**Fig. 2:**
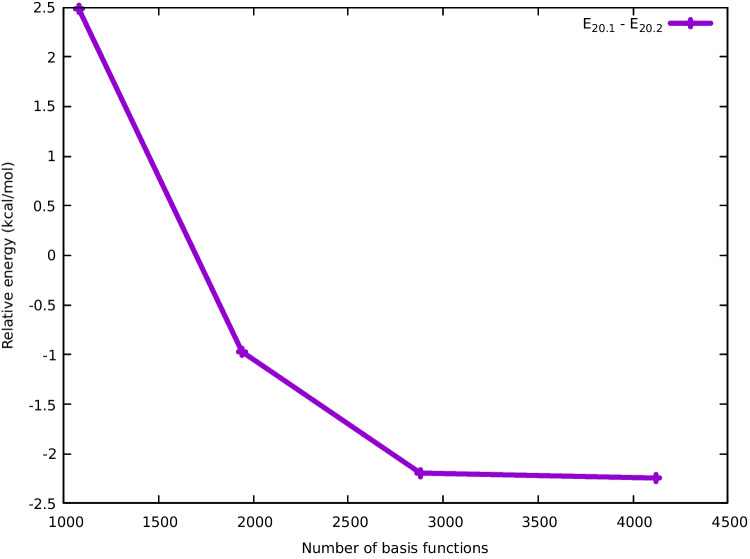
Relative energy of the **20**.**1** cluster with respect to **20**.**2** as a function of the number of basis set functions. All calculation were done using the TPSSh functional

### On the role of the basis set

Besides the importance of selecting the correct functional approximation, the selection of basis sets is of paramount importance for the accurate assignment of the energy ordering in these systems. Figure 2 illustrates the relative energy of the **20**.**1** cluster as compared to **20**.**2** for different basis sets taken from the cc-pwCVXZ-PP family  [36]. We observe that the smallest basis set, cc-pwCVDZ-PP with 1080 functions, places **20**.**1** higher in energy than **20**.**2**. However, when the cc-pwCVTZ-PP basis set is considered, **20**.**1** is correctly identified as the true GM. Notably, this behavior is not oscillatory, and the selection of **20**.**1** as the GM is confirmed by the larger cc-pwCVQZ-PP and cc-pwCV5Z-PP basis sets, which have 2880 and 4120 functions, respectively. These results suggest that the use of basis sets of at least QZ quality is essential for the accurate differentiation of conformers with similar energies.

Table 2 illustrates the relative energies of the **20**.**1** and **20**.**2** isomers using different functionals and basis sets of triple and quadruple-zeta quality. In particular, we selected the functionals mentioned above (PBE, PBE0, TPSS, TPSSh, and $\omega$B97x) and included also the popular BP86 [37, 38] and B3PW91 [39, 40] approximations in combination with the Def2-TZVP (TZ) and Def2-QZVPP (QZ) basis sets. Remarkably, older GGA functionals (BP86 and PBE) exhibited a pronounced preference for **20**.**2** over **20**.**1**, with energy differences of 9.8 and 7.9 kcal/mol, respectively. Moreover, the incorporation of exact exchange has a clear stabilizing effect on **20**.**1** relative to **20**.**2**: while PBE/TZ favored **20**.**2** by 7.9 kcal/mol, PBE0/TZ favored it only by 5.2 kcal/mol, a difference of 2.7 kcal/mol. In contrast, the newer $\omega$B97x, a range-separated hybrid functional, favored the **20**.**1** isomer by 4.2 kcal/mol.

**Table 2:** Relative energies (kcal/mol) of the two lowest-lying isomers of Pt20 computed with different exchange-correlation functionals and basis sets. In the last column, the indicated quantities are $\Delta$E(QZ) - $\Delta$E(TZ) where $\Delta$E(QZ)= $\Delta$E$_{20.2}$(QZ) - E$_{20.1}$(QZ). Ditto for $\Delta$E(TZ)

Functional	20.1	20.2$^{a}$	$\Delta$E(QZ) - $\Delta$E(TZ)
$\omega$B97x/TZ	0.0	4.1	
$\omega$B97x/QZ	0.0	9.2	5.1
TPSSh/TZ	4.4	0.0	
TPSSh/QZ	0.0	1.1	5.5
TPSS/TZ	4.4	0.0	
TPSS/QZ	0.0	0.7	5.1
PBE0/TZ	5.2	0.0	
PBE0/QZ	0.0	0.0	5.2
PBE/TZ	7.9	0.0	
PBE/QZ	3.1	0.0	4.8
B3PW91/TZ	7.8	0.0	
B3PW91/QZ	3.0	0.0	4.8
BP86/TZ	9.8	0.0	
BP86/QZ	5.2	0.0	4.6

$$^{\text {a}}$$ Ref. [18]

**Fig. 3:**
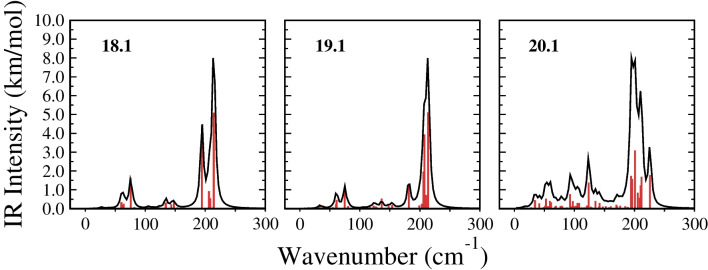
IR spectra for the lowest energy structures of $\text {Pt}_{\textrm{n}}$ ($\textrm{n}=18$, 19, 20) clusters at the TPSSh/Def2TZVP level of theory

It is striking that the transition from TZ to QZ-quality basis sets has significant implications for all functionals, irrespective of whether they are GGA, meta-GGA, or hybrid. Using the PBE functional and the TZ basis sets, **20**.**2** is 7.9 kcal/mol more stable than **20**.**1**, while using the QZ basis reduces this difference to only 3.1 kcal/mol. Even more importantly, the use of QZ basis sets is cabable of reversing the energetic ordering, as observed with the TPSS and TPSSh functionals. For TPSS/TZ, **20**.**2** is the GM, while it is **20**.**1** in the case of TPSS/QZ. Indeed, going from the TZ to the QZ basis sets favors **20**.**1** over **20**.**2** by approximately 5 kcal/mol, regardless of its reference value. This is observed in the last column of Table 2. From these observations, we can infer that basis sets incompleteness has a selective impact on the relative stability of different isomers. Furthermore, these findings confirm the importance of using high-quality basis sets to obtain meaningful results for relative energies and other properties  [31]. Our results emphasize the importance of selecting an appropriate functional approximation and a sufficiently large basis set to ascertain the relative stability of metal clusters in general and $\text {Pt}_{\textrm{n}}$ isomers in particular.

**Fig. 4:**
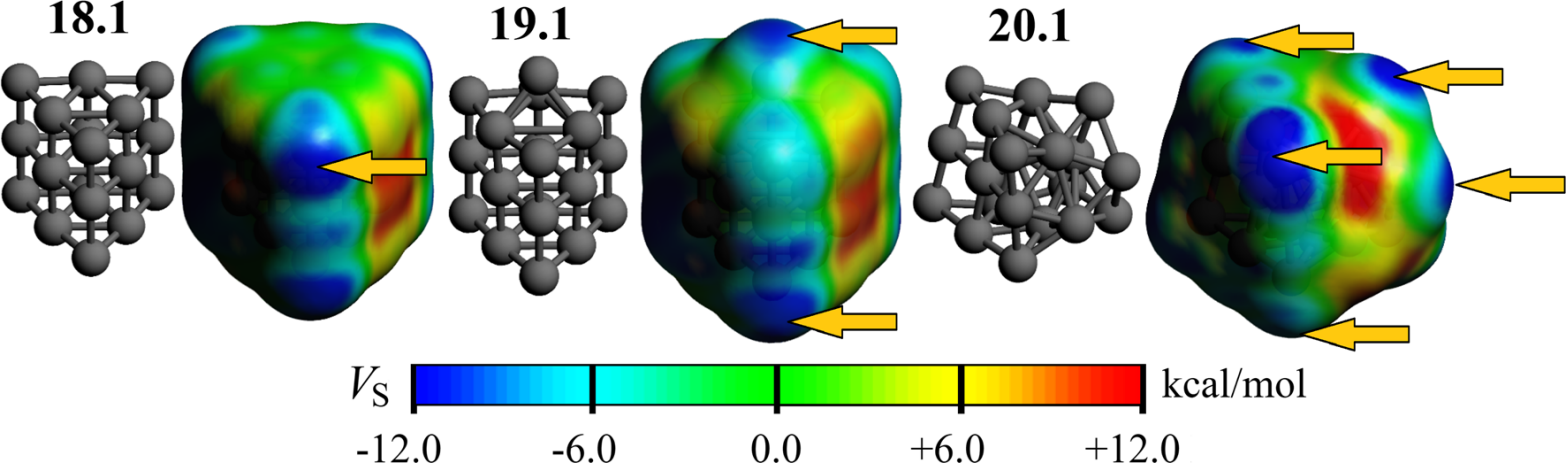
Electrostatic potential ($V_{\textrm{S}}$) mapped onto the van der Waals envelope for the minimum energy structures of Pt$_{18}$, Pt$_{19}$, and Pt$_{20}$. $\sigma$-holes, identified as maximally negative $V_{\textrm{S}}$ sites, are highlighted by yellow arrows. See the text for further details

**Fig. 5:**
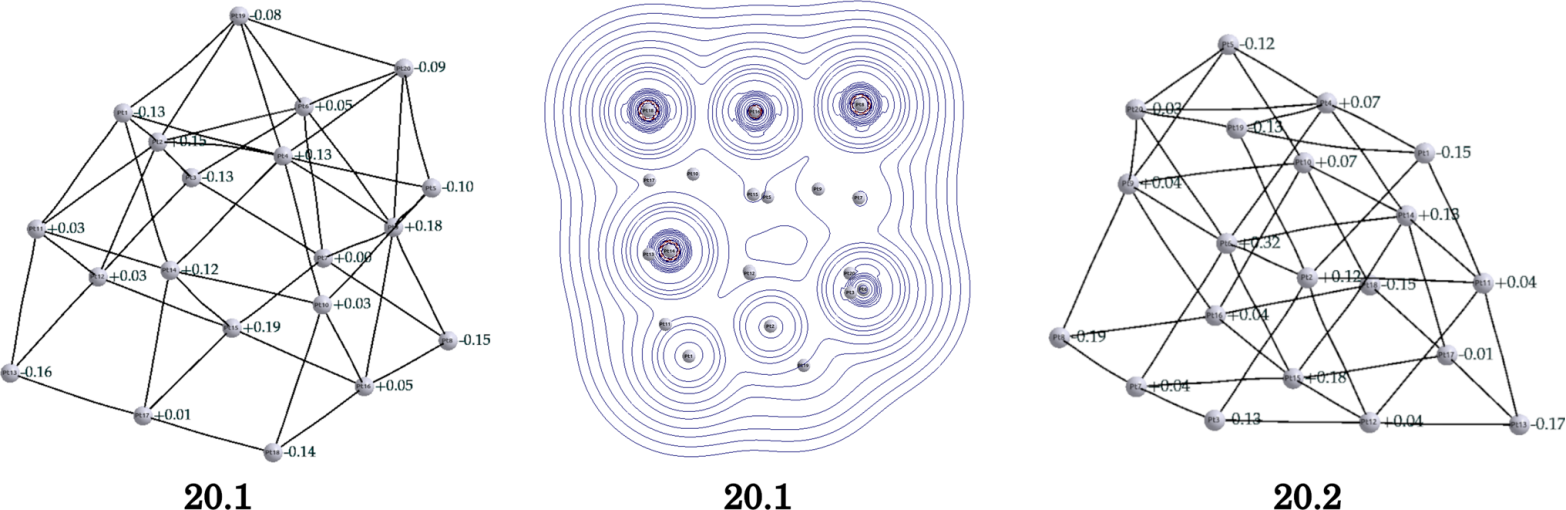
Bond paths and atomic charges for the minima structures of the **20**.**1** and **20**.**2** clusters. Two-dimensional relieve map of the Laplacian of the electronic density for the **20**.**1** cluster plotted in the plane formed by the Pt8, Pt14, and Pt18 atoms

### IR fingerprints

In order to provide fingerprints for structural identification, we have calculated the vibrational modes of the most stable clusters. The characteristic peaks for Pt$_n$ (n=18, 19, 20) clusters were found at 194.3, 207.6, and 200.8 cm$^{-1}$, respectively. These correspond to the antisymmetric stretching of the central layers. The lowest vibrational frequencies, observed at 27.1, 25.7, and 16.8 cm$^{-1}$, are twisting vibrations, while the vibrations at the highest frequencies are located at 214.0, 213.7, and 225.6 cm$^{-1}$, are antisymmetric. The IR spectra of these clusters were generated using the orca_mapspc utility within the ORCA program and are shown in Fig. 3. These spectra could serve as benchmarks for future experimental characterization of Pt clusters.

### Electrostatic potentials and QTAIM analyses

Theoretical studies on the lowest energy structures of Pt clusters provide insight into the underlying mechanisms that contribute to their excellent catalytic performance, as observed in experiments [24]. The overall catalytic efficiency of a given aggregate can be related to the existence of specific catalytically active sites, which are determined by the location of low-coordinated atoms within the cluster structure. As observed in gold and other noble metals, such sites lead to electron-deficient regions, the so-called $\sigma$-holes, that can be evaluated via the electrostatic potential mapped onto the van der Waals envelope (conveniently approximated as an electron density isosurface with isovalue 0.001 a.u.) [41, 42].

As illustrated in Fig. 4, the resulting electrostatic potential surface indicates the formation of six equivalent low-coordinated edges in Pt$_{18}$, which give rise to six analogous $\sigma$-holes. These pinpoint reactive sites, as evidenced by the negative values of the electrostatic potential ($V_{\textrm{S}}$). The addition of a Pt atom to form Pt$_{19}$ results in three equivalent $\sigma$-holes that retain some characteristics of the Pt$_{18}$ parent, together with the appearance of a new deep hole around the capping atom. We thus expect different reactive sites in different regions of the cluster. This is exacerbated in Pt$_{20}$, where the marked decrease in symmetry gives rise to several different $\sigma$-hole sites. Since the number of catalytic active regions in this cluster is greater than that observed in Pt$_{18}$ and Pt$_{19}$, we conclude that this cluster will be particularly reactive. Hence, the controlled growth of these types of aggregates may prove an effective strategy for modifying the number of catalytic sites within a narrow size range.

The Quantum Theory of Atoms in Molecules (QTAIM) is a powerful tool for studying chemical interactions within a molecule. It allows for an orbital invariant analysis of electron density, thereby providing insights into the nature and strength of interactions between atoms. The QTAIM has been successfully employed in the study of intermetallic interactions [43–45] and metallic clusters [46–51]. Figure 5 provides such an analysis for the **20**.**1** and **20**.**2** clusters, illustrating both the bond paths [52, 53] between the platinum atoms and their atomic charges  [54]. The electron densities of these two clusters are strikingly similar, exhibiting concentration of charge at the edge atoms coupled with a depletion of density in the interior atoms, which have a larger number of immediate neighbors. This is in contrast with previous findings in Al$_{\textrm{n}}$Sc clusters, where the electron density was found to be larger for the endohedral atom [51]. Furthermore, Fig. 5 also shows a two-dimensional relief map of the Laplacian of the electron density. This dissection reveals charge depletion zones that coincide with the catalytic active areas depicted in Fig. 4. This observation suggests a potential link between the distribution of the electron density near the nucleus and the catalytic properties of these Pt clusters, which merits further investigation.

## Methods

We employed a machine-learning interatomic potential constructed from DFT energies and forces calculated at the TPSS/Def2-TZVP level of theory in order to ease the mapping of the complex potential energy surface of the Pt$_{20}$ clusters. This potential was developed using E(3)-equivariant graph neural networks within the NequIP program by Batzner and coworkers  [55], and it was used in conjunction with the Atomic Simulation Environment suite  [56]. We used this interatomic potential to perform simulated annealing minimisations based on molecular dynamics simulations. The resulting structures were reoptimised using different exchange-correlation functionals, namely BP86  [37, 38], PBE  [57], PBEh  [58], PBE0  [59], B3PW91  [39, 40], TPSS  [60], TPSSh [61], M06-L  [62], and $\omega$B97x  [63], in combination with the Def2-TZVP (TZ) and Def2-QZVP (QZ)  [64–66] basis sets which include a relativistic pseudopotential replacing 60 core electrons of the platinum atom. With the aim of calibrating the performance of DFT methods, in our previous work, we conducted a benchmark calculation for Pt$_2$ dimers described by different types of functionals [23]. By comparing the ionization potential and dissociation energies of these clusters, the results indicated that the hybrid-GGA M06-L, B3PW91, and meta-GGA TPSS functionals were good choices to evaluate the structures of the clusters. Interestingly, the TPSSh functional provided a bond length of 2.34 Å, which is slightly smaller than that predicted by TPSS (with 2.35 Å), but in better agreement with the experiment of Airola and Morse [67] (2.33 Å). Thus, the TPSSh functional seems realiable in predicting molecular geometries and vibrational frequencies for these systems. All DFT calculations were conducted with the aid of the Orca 5 software [68]. For the QTAIM [54] analyses, densities were obtained using the Zeroth-Order Regular Approximation [69–71], and the density partitions themselves were carried out using the AIMAll program  [72]. The resulting structures were visualized using the Avogadro code  [73].

## Conclusion

In this study, we employed Density Functional Theory (DFT) to investigate the lowest energy structures and electronic properties of Pt$_{18}$, Pt$_{19}$, and Pt$_{20}$ clusters. Our findings have revealed the existence of novel, more stable isomers for the Pt$_{19}$ and Pt$_{20}$ systems, 3.0 and 1.0 kcal/mol more stable, respectively, than the previously reported minimum energy structures. Furthermore, the role of different DFT approximations, including GGA (PBE), meta-GGA (TPSS, M06-L), hybrid (PBE0), meta-GGA hybrid (TPSSh), and range-separated hybrid ($\omega$B97x) functionals, was investigated in relation to the relative energies of the studied clusters. Our findings indicate that the energy ordering of different isomers is highly sensitive to the use of density functional approximations that include some exact Hartree-Fock exchange. Furthermore, it is evident that the use of basis sets of at least quadruple-zeta quality is necessary. A QTAIM analysis highlights significant distinctions in both the electron distribution and the nature of interatomic contacts among isomers, even those with comparable total energies. The MEP of the novel Pt$_{20}$ minimum indicates the existence of potential catalytic sites. The insights presented in this article are expected to facilitate the design of future simulations and contribute to the accurate energetic ordering of isomers of metallic clusters, ultimately enabling the identification of true global minima in noble metal clusters as prototypical catalytic reactive species.

## Supplementary information

A supplementary file with the structures of the studied clusters is available.

## Supplementary Information

Below is the link to the electronic supplementary material.

Supplementary file 1 (pdf 121 KB)

## Data Availability

No datasets were generated or analyzed during the current study.
