# Supplementary material for: New global minimum conformers for the Pt19 and Pt20 clusters: low symmetric species featuring different active sites
Source: J Mol Model. 2024 Aug 17;30(9):310. doi: 10.1007/s00894-024-06099-5 (PMC11330413; doi:10.1007/s00894-024-06099-5)
Supplement: Supplementary file 1 — (pdf 121 KB) [file 894_2024_6099_MOESM1_ESM.pdf]

New global minimum conformers for the Pt_{19} and Pt_{20} clusters. Low symmetric species featuring different active sites.

José Manuel Guevara-Vela[a], Miguel Gallegos[b], Tomás Rocha-Rinza[c], Álvaro Muñoz-Castro[d], Peter L. Rodríguez-Kessler[e,*], Ángel Martín Pendás[b,**]

[a]*Departamento de Química Física Aplicada. Universidad Autónoma de Madrid, Madrid 28049, Spain.*
[b]*Departamento de Química Física y Analítica. Universidad de Oviedo. 33006, Oviedo, Spain.*
[c]*Instituto de Química, Universidad Nacional Autónoma de México, Circuito Exterior, Ciudad Universitaria, Delegación Coyoacán C.P. 04510, Ciudad de Miéxico, Mexico.*
[d]*Facultad de Ingeniería, Arquitectura y Diseño, Universidad San Sebastián, Bellavista 7, Santiago, 8420524, Chile.*
[e]*Centro de Investigaciones en Óptica A.C., Loma del Bosque 115, Col. Lomas del Campestre, León, Guanajuato, Mexico.*

*To whom correspondence should be addressed: plkessler@cio.mx
**To whom correspondence should be addressed: ampendas@uniovi.es

Table S1: Atomic coordinates for the **18.1** cluster at the TPSSh/Def2-TZVP level of theory.

Atom	X	Y	Z
Pt	-1.76766	-3.11134	0.00202
Pt	-2.14501	-1.00983	1.37010
Pt	-2.53075	1.21081	2.53369
Pt	0.70787	-2.65665	-0.01657
Pt	3.18621	-2.20609	-0.03532
Pt	0.34414	-0.59655	1.42191
Pt	-0.05425	1.66146	2.51454
Pt	2.42407	2.11456	2.49751
Pt	2.81904	-0.10400	1.33316
Pt	2.79805	-0.10722	-1.40302
Pt	-2.16573	-1.01384	-1.36640
Pt	-2.57323	1.32065	0.00157
Pt	-2.56814	1.20348	-2.53044
Pt	0.32224	-0.60006	-1.45448
Pt	-0.10650	1.85331	-0.01661
Pt	2.39119	2.22647	-0.03503
Pt	-0.09176	1.65471	-2.54726
Pt	2.38611	2.10788	-2.56665

Table S2: Atomic coordinates for the **18.2** cluster at the TPSSh/Def2-TZVP level of theory.

Atom	X	Y	Z
Pt	-1.89049	-3.07747	0.00105
Pt	-2.13936	-1.23913	1.75711
Pt	-2.51678	1.19519	2.48944
Pt	0.61716	-2.77635	-0.01068
Pt	3.10307	-2.37780	-0.02281
Pt	0.31618	-0.73065	1.47274
Pt	-0.01677	1.52080	2.56838
Pt	2.46285	1.96171	2.54090
Pt	2.77043	-0.25598	1.34821
Pt	2.75727	-0.24940	-1.38012
Pt	-2.15511	-1.23304	-1.74601
Pt	-2.39355	0.53184	0.00988
Pt	-2.53973	1.20402	-2.46609
Pt	0.30236	-0.72466	-1.48273
Pt	-0.05384	1.67685	0.00172
Pt	2.44547	2.13599	-0.00826
Pt	-0.04123	1.53132	-2.56576
Pt	2.43747	1.97437	-2.55827

Table S3: Atomic coordinates for the **18.3** cluster at the TPSSh/Def2-TZVP level of theory.

Atom	X	Y	Z
Pt	1.24718	0.80398	-0.00468
Pt	-0.68111	2.46105	0.07258
Pt	0.60137	-0.13821	2.28902
Pt	-0.96963	1.82723	2.49488
Pt	0.01225	-0.92360	4.65392
Pt	-1.18903	1.28995	4.94387
Pt	0.40237	3.61397	5.03583
Pt	0.19220	4.46323	2.67269
Pt	-0.04639	5.20188	0.27865
Pt	1.88611	3.31657	-0.02024
Pt	1.89772	2.15667	2.31900
Pt	1.51300	1.16676	4.70328
Pt	-2.42606	4.30910	0.30918
Pt	-2.29798	3.97199	2.80672
Pt	-2.13613	3.62049	5.30062
Pt	2.40535	5.83210	0.14189
Pt	2.69405	4.66781	2.40376
Pt	2.89475	3.35903	4.59901

Table S4: Atomic coordinates for the **19.1** cluster at the TPSSh/Def2-TZVP level of theory.

Atom	X	Y	Z
Pt	-2.74171	2.72957	1.06713
Pt	-2.74344	1.23626	-0.99076
Pt	-2.73859	-0.44083	-2.90068
Pt	-0.21698	2.72870	1.06435
Pt	2.29401	2.71891	1.05894
Pt	-0.21643	1.27889	-1.02377
Pt	-0.21431	-0.44151	-2.89449
Pt	2.29639	-0.44018	-2.87880
Pt	2.26576	1.42743	-1.14098
Pt	2.26736	0.27930	1.80827
Pt	-2.74266	0.23652	1.56654
Pt	-2.74172	-1.47729	-0.57851
Pt	-2.73762	-2.29161	1.83062
Pt	-0.21536	0.24785	1.62032
Pt	-0.21481	-1.52716	-0.59622
Pt	2.26696	-1.70629	-0.66475
Pt	-0.21299	-2.28473	1.83009
Pt	2.29746	-2.27163	1.82286
Pt	4.04867	-0.00219	-0.00018

Table S5: Atomic coordinates for the **19.2** cluster at the TPSSh/Def2-TZVP level of theory.

Atom	X	Y	Z
Pt	2.95168	-1.11870	-2.53465
Pt	2.64471	1.12563	-1.36725
Pt	2.34907	3.24289	0.00184
Pt	0.46271	-1.49714	-2.53564
Pt	-2.01939	-1.89552	-2.55093
Pt	0.12332	0.79830	-1.45900
Pt	-0.16888	2.83869	0.00237
Pt	-2.67419	2.45829	0.00294
Pt	-2.36386	0.37804	-1.50702
Pt	-2.06012	-1.78903	-0.00056
Pt	2.95097	-1.24136	-0.00216
Pt	2.64578	1.12330	1.36709
Pt	2.95368	-1.12290	2.53040
Pt	0.46332	-1.61794	-0.00143
Pt	0.12446	0.79590	1.46017
Pt	-2.36273	0.37566	1.50941
Pt	0.46468	-1.50114	2.53283
Pt	-2.01746	-1.89938	2.54950
Pt	-4.46775	0.54640	0.00209

Table S6: Atomic coordinates for the **19.3** cluster at the TPSSh/Def2-TZVP level of theory.

Atom	X	Y	Z
Pt	-4.01021	0.00474	-0.57760
Pt	-2.56019	1.89630	0.28010
Pt	-0.87521	3.23426	1.63849
Pt	-1.41056	-3.28047	-0.94849
Pt	-0.62757	1.22425	-1.21187
Pt	-2.05736	-0.67025	-2.06970
Pt	-1.19985	0.72387	2.14781
Pt	-1.29972	-1.75618	2.89555
Pt	1.72554	-1.03535	-0.81491
Pt	0.11943	-1.85551	-2.60909
Pt	-1.91261	-1.12708	0.40173
Pt	0.84035	3.00797	-0.21802
Pt	2.66273	2.43907	-1.87167
Pt	1.23300	0.48600	-2.74788
Pt	1.15345	0.91228	1.16570
Pt	1.28918	-1.13656	2.68086
Pt	3.36861	0.74308	-0.10529
Pt	3.32553	-1.45807	1.19077
Pt	0.23544	-2.35235	0.77349

Table S7: Atomic coordinates for the **20.1** cluster at the TPSSh/Def2-TZVP level of theory.

Atom	X	Y	Z
Pt	-0.87538	-3.25234	-0.29410
Pt	-0.62709	-1.53417	1.57019
Pt	-0.44405	0.27637	3.41137
Pt	1.15174	-1.47911	-0.68121
Pt	3.11940	-0.51961	-2.11561
Pt	1.73446	-0.59665	2.26282
Pt	0.93459	1.87148	1.92622
Pt	2.19306	3.40424	0.35580
Pt	2.22895	0.83778	-0.16021
Pt	0.70512	0.20979	-2.74216
Pt	-2.97915	-1.76009	0.21603
Pt	-2.47447	0.34492	1.82003
Pt	-4.39897	0.36252	0.14419
Pt	-1.29581	-0.87067	-1.41202
Pt	-0.85328	1.38696	0.16236
Pt	0.69532	2.52600	-1.51489
Pt	-2.72844	1.22056	-1.58017
Pt	-1.05768	1.95408	-3.31468
Pt	1.46467	-3.05421	1.39971
Pt	3.50703	-1.32786	0.54634

Table S8: Atomic coordinates for the **20.2** cluster at the TPSSh/Def2-TZVP level of theory.

Atom	X	Y	Z
Pt	-1.90645	-3.17541	-0.26353
Pt	-1.93376	-1.17745	1.32689
Pt	-2.24623	0.98705	2.61511
Pt	0.54529	-2.59052	0.01152
Pt	3.01647	-2.14251	0.30847
Pt	0.46704	-0.31567	1.27532
Pt	0.14257	1.86162	2.52454
Pt	2.59872	2.50366	2.42320
Pt	2.92958	0.10783	1.58464
Pt	2.68624	-0.12042	-1.23814
Pt	-2.26812	-0.99252	-1.54240
Pt	-2.53283	1.11236	0.08859
Pt	-2.64153	1.34718	-2.46229
Pt	0.24001	-0.64289	-1.60940
Pt	-0.07274	1.84955	-0.04070
Pt	2.44865	2.28921	-0.11516
Pt	-0.13600	1.66431	-2.59617
Pt	2.36748	1.96747	-2.64754
Pt	-0.76025	-3.18156	2.35387
Pt	1.61017	-2.09985	2.61421

Table S9: Atomic coordinates for the **20.3** cluster at the TPSSh/Def2-TZVP level of theory.

Atom	X	Y	Z
Pt	-2.44089	-0.69974	0.02783
Pt	-1.66795	-3.11465	0.28871
Pt	-2.63990	1.81459	0.34625
Pt	-0.16959	2.35053	-0.04423
Pt	0.01690	-0.23786	0.00378
Pt	0.32643	-2.17098	1.64851
Pt	1.44743	2.49957	1.97847
Pt	1.97032	-0.28885	2.46849
Pt	-0.35251	0.71653	2.43854
Pt	-2.82451	1.21967	2.80462
Pt	-2.04000	-1.24888	2.48227
Pt	2.51661	-0.34371	-0.00929
Pt	2.09791	-2.85436	-0.17584
Pt	2.35327	2.15953	-0.41930
Pt	-0.00611	-2.25563	-1.56475
Pt	-1.78590	2.18892	-2.06623
Pt	-1.90198	-0.66358	-2.45142
Pt	0.25228	0.66568	-2.46627
Pt	2.62817	1.50524	-2.85299
Pt	2.20526	-1.03673	-2.44102
